# Population genomics and subgenome evolution of the allotetraploid frog *Xenopus laevis* in southern Africa

**DOI:** 10.1093/g3journal/jkac325

**Published:** 2022-12-16

**Authors:** Tharindu Premachandra, Caroline M S Cauret, Werner Conradie, John Measey, Ben J Evans

**Affiliations:** Department of Biology, McMaster University, Life Sciences Building Room 328, 1280 Main Street West, Hamilton, ON L8S4K1, Canada; Department of Biology, McMaster University, Life Sciences Building Room 328, 1280 Main Street West, Hamilton, ON L8S4K1, Canada; Department of Botany and Plant Pathology, Oregon State University, Corvallis, OR 97331, USA; Port Elizabeth Museum (Bayworld), P.O. Box 13147, Humewood, Gqeberha 6013, South Africa; Department of Conservation Management, Natural Resource Science and Management Cluster, Faculty of Science, Nelson Mandela University, George Campus, George 6019, South Africa; Centre for Invasion Biology, Department of Botany and Zoology, Stellenbosch University, Stellenbosch 7602, South Africa; Department of Biology, McMaster University, Life Sciences Building Room 328, 1280 Main Street West, Hamilton, ON L8S4K1, Canada

**Keywords:** gene flow, phylogeography, polyploid ratchet, population genetics

## Abstract

Allotetraploid genomes have two distinct genomic components called subgenomes that are derived from separate diploid ancestral species. Many genomic characteristics such as gene function, expression, recombination, and transposable element mobility may differ significantly between subgenomes. To explore the possibility that subgenome population structure and gene flow may differ as well, we examined genetic variation in an allotetraploid frog—the African clawed frog (*Xenopus laevis*)—over the dynamic and varied habitat of its native range in southern Africa. Using reduced representation genome sequences from 91 samples from 12 localities, we found no strong evidence that population structure and gene flow differed substantially by subgenome. We then compared patterns of population structure in the nuclear genome to the mitochondrial genome using Sanger sequences from 455 samples from 183 localities. Our results provide further resolution to the geographic distribution of mitochondrial and nuclear diversity in this species and illustrate that population structure in both genomes corresponds roughly with variation in seasonal rainfall and with the topography of southern Africa.

## Introduction

Whole-genome duplication (or polyploidization) can occur spontaneously within a species (autopolyploidization) or in association with hybridization among different species (allopolyploidization). Polyploidization preceded extraordinary diversifications such as eudicot plants, jawed vertebrates, and teleost fish, and more recently in many species of agricultural and scientific importance (wheat, corn, cotton, rice, yeast, paramecium, tetrahymena; Otto and Whitton 2000; Van de Peer *et al*. 2009; Bomblies 2020; Lovell *et al*. 2021). This raises the questions of how whole-genome duplication influences adaptation, diversification, and genome evolution.

Polyploid genomes are a combination of at least two ancestral genomes from one (autopolyploids) or two (allopolyploids) ancestral species. Each of these ancestral genomes forms a subgenome in the new polyploid genome. A polyploid individual may have polysomic inheritance, where chiasmata form between more than two homologous chromosomes. In a tetraploid species with tetrasomic inheritance, for example, chiasmata can involve four chromosomes, including one pair from each subgenome. Polysomic inheritance may be associated with chromosomal mis-segregation, which could favor the evolution of diploid-like (disomic) gametogenesis, wherein each chromosome pairs with only one homologous chromosome during meiosis I (Soares *et al*. 2021). Disomic inheritance can be achieved instantly upon polyploidization or gradually over time if recombination becomes rare or ceases between each subgenome. Rapid evolution of disomic inheritance may be more common in allopolyploids than autopolyploids owing to the more pronounced divergence between orthologous pairs of homeologous chromosomes in allopolyploids (Wolfe 2001). Further complexity arises when subgenomes establish disomic inheritance at different times: prior to the origin of disomic inheritance, genetic drift or natural selection can contribute to the elimination of variation from one ancestral species in portions of the genome with polysomic inheritance (Wolfe 2001). However, genetic exchanges between subgenomes such as translocation, exchange of transposable elements, and recombination events such as gene conversion may occur. For example, in the Moscow salsify plant (*Tragopogon miscellus*), translocations between subgenomes are relatively common (Chester *et al*. 2012). Similarly, in the allotetraploid African clawed frog (*Xenopus laevis*), a sex determining gene called *dm-W* resides in one subgenome (the L subgenome), but is derived from a partial duplication of a gene (*dmrt1S*) that resides in the other subgenome (the S subgenome; Bewick *et al*. 2011).

Subgenomes may evolve distinctive features such as differences in gene expression, gene silencing, alternative splicing, rates of recombination and rearrangement, rates of protein evolution, and complements and mobilities of transposable elements and other repetitive elements (Liu *et al*. 2014; Session *et al*. 2016; Elurbe *et al*. 2017; Mei *et al*. 2017; Cheng *et al*. 2018; Furman *et al*. 2018; Edger *et al*. 2019; Zhao *et al*. 2020; Jia *et al*. 2021; Schiavinato *et al*. 2021). Subgenome-specific genomic changes can have fitness consequences, such as centromere homogenization and distorted segregation, that create a downward fitness spiral called the “polyploid ratchet” where genomic instability begets further instability (Gaeta and Pires 2010). Evidence for the polyploid ratchet is recovered from comparisons between natural and synthetic polyploids: rearrangements in synthetic allopolyploids are frequently more common than in (older) natural allopolyploids, suggesting the action of purifying selection and other processes that increase genomic stability over time (Gaeta *et al*. 2007). Subgenome-specific gene flow (between populations or species) has the potential to counteract the polyploid ratchet by restoring genetic variation that was lost or damaged during allopolyploid evolution. In allotetraploid barbed goatgrass (*Aegilops triuncialis*), for example, gene flow with domestic wheat is higher in one subgenome compared with the other, which is possibly linked to variation between subgenomes in genomic stability and gene content (Parisod *et al*. 2013). Differential gene flow in each subgenome also has potential phenotypic consequences. In various plants from the agriculturally important genus *Brassica*, several allotetraploids are derived from different combinations of three diploid progenitors; subgenome-specific introgression between these allotetraploids and other synthetic polyploids corresponds with variation in phenotypes (Wei *et al*. 2016). Asymmetry in subgenome evolution has the potential to be influenced by natural selection on mitonuclear interactions because the mitochondrial genome is inherited from only one of the two ancestral species of an allopolyploid genome (Sharbrough *et al*. 2021). For example, because they share a longer co-evolutionary history, natural selection may favor autosomal genes encoded by the subgenome that is derived from the same ancestor as the mitochondrial genome when compared with homeologous autosomal loci from the other subgenome.

**Fig. 1.**
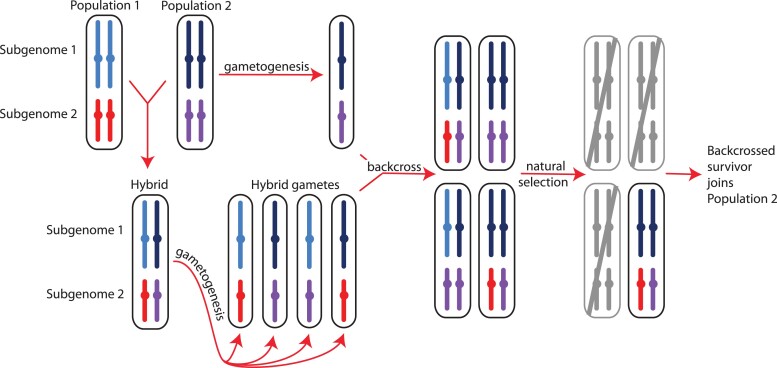
Homoploid hybridization—gene flow between individuals with the same ploidy level (which in this example are both polyploid individuals)—can lead to differential introgression in subgenomes, either due to random events or in association with natural selection against some admixed genotypes as shown above. The parental populations (populations 1 and 2) are assumed to have two subgenomes with disomic inheritance (indicated with light blue and dark blue or red and purple, respectively, with color differences within each subgenome representing population subdivision). Gametogenesis in a hybrid individual produces gametes with a mosaic of population variation in each subgenome, depicted here as blocks that include entire chromosomes (in reality, recombination would likely generate smaller blocks of population-specific variation within each chromosome). If some backcrossed individuals have low fitness (gray) and high fitness individuals successfully breed in one parental population, different proportions of each subgenome may introgress, here shown as an extreme example of introgression only in subgenome 2.

How might gene flow differ between subgenomes? This can be illustrated by considering a hypothetical example of “homoploid” hybridization, wherein gene flow occurs between two parental allotetraploid populations that both have disomic inheritance (Fig. 1). The hybrid progeny of a cross between non-admixed parental individuals from each population is expected to also have disomic inheritance and would be heterozygous in both subgenomes for population-specific variation from each parental population. A gamete of this hybrid individual thus would carry a mosaic of alleles from each parental population, with 50% probability of carrying a population-specific allele from either one. If backcrossed to an individual from one parental population, the resulting backcrossed individuals would have a mosaic of homozygous and heterozygous genotypes from both parental populations. This genetic variation among siblings is subject to natural selection and genetic drift, which could result in asymmetry in the extent of introgression in each subgenome if backcrossed survivors join a parental population (an example of more gene flow in subgenome 2 than subgenome 1 is depicted in Fig. 1).

### Allopolyploid evolution in African clawed frogs

African clawed frogs (genus *Xenopus*) underwent at least two independent allotetraploidization, three independent allooctoploidization, and three independent allododecaploidization events, which gave rise to a striking diversity of polyploid species: 13 allotetraploids, seven allooctoploids, at least three allododecaploids (Evans 2008; Evans *et al.* 2015). A probable mechanism for allopolyploidization involves successive generations of backcrossing and production of unreduced gametes by hybrid females, and has been recapitulated in crosses of captive animals (Kobel and Du Pasquier 1986). Polyploidization in *Xenopus* is thought to involve unreduced female eggs rather than unreduced sperm because at least some hybrid males may be sterile (Kobel 1996), an observation that goes against expectations of Haldane's (1922) Rule because females are heterogametic in most *Xenopus*.

The African clawed frog *X. laevis* is a widely used model organism for biology (Cannatella and de Sá 1993; Gurdon and Hopwood 2000) and a high-quality genome assembly is available (Session *et al*. 2016). The distribution of *X. laevis* spans multiple biomes including (from southwest to northeast): fynbos, succulent Karoo, Nama-Karoo, forest, thicket, savanna, and grassland (Mucina and Rutherford 2006), and patterns of population structure roughly correspond with geographic variation in annual rainfall patterns with winter rainfall in the southwest and summer rainfall in the northeast (Grohovaz *et al.* 1996; Evans *et al.* 1997; Measey and Channing 2003; Evans *et al.* 2004; Du Preez *et al.* 2009; Furman *et al.* 2015). Phenotypic variation exists within *X. laevis*, such as size differences (larger in the SW) and variation in gonadal morphology, with a higher frequency of testicular ovarian follicles detected in animals originating from northeast compared with southwest South Africa (Du Preez *et al*. 2009). Reciprocal translocation experiments suggest adaptation to different rainfall and altitudinal regimes, as well as variation in the extent of plasticity of tadpoles to adjust to these regimes (Wagener *et al*. 2021; Kruger *et al*. 2022). Within *X. laevis*, mitochondrial DNA is paraphyletic, and considerable population structure exists, especially in the southwest Cape Region on either side of the Cape Fold Mountains (Grohovaz *et al.* 1996; Evans *et al.* 1997; Measey and Channing 2003; Evans *et al.* 2004; Du Preez *et al.* 2009; Furman *et al.* 2015).

In *X. laevis*, which is an allotetraploid species, subgenomes are karyotypically distinguished by distinctive sizes of homeologous chromosomes, such that the L and S subgenomes have long and short versions of homeologous chromosomes (Matsuda *et al*. 2015; Session *et al*. 2016). A striking finding that emerged from analysis of a high-quality, chromosome-scale genome sequence of *X. laevis* (Session *et al*. 2016; Elurbe *et al*. 2017), and other allotetraploid *Xenopus* (Furman *et al*. 2018) is that each subgenome also has distinctive molecular genetic characteristics, with the L subgenome encoding transcripts that have a lower rate of gene silencing, higher expression level, stronger purifying selection, and longer coding regions compared with homeologous transcripts from the S subgenome. Additionally, the S subgenome experienced more rearrangements than the L subgenome (Session *et al*. 2016).

We had an a priori expectation that, across the native habitats of *X. laevis*, the L subgenome would have more geographic structure (less gene flow) than the S subgenome. This expectation derives from (1) previous observations of stronger purifying selection on genes (Furman *et al*. 2018) and a lower rate of pseudogenization (Session *et al*. 2016) in the L subgenome, presumably including some genes that are involved with adaptation to local ecological conditions, and (2) previous observations of higher genomic instability in the S subgenome (Session *et al*. 2016), which could be associated with the polyploid ratchet and potentially mitigated by gene flow. To test this expectation, we set out to further characterize genetic variation in each *X. laevis* subgenome in its natural range with a focus on southwestern South Africa where most of the genetic variation occurs, including samples within and on either side of an intraspecific contact zone (Du Preez *et al*. 2009; Furman *et al*. 2015) that corresponds to changes in both biome and rainfall (Chase and Meadows 2007). We also compared patterns of genetic variation in each subgenome based on reduced representation genome sequencing (RRGS) data to that in the mitochondrial genome based on Sanger sequences from an even more widespread sample of wild caught animals.

## Methods

### Samples and genetic data

**Fig. 2.**
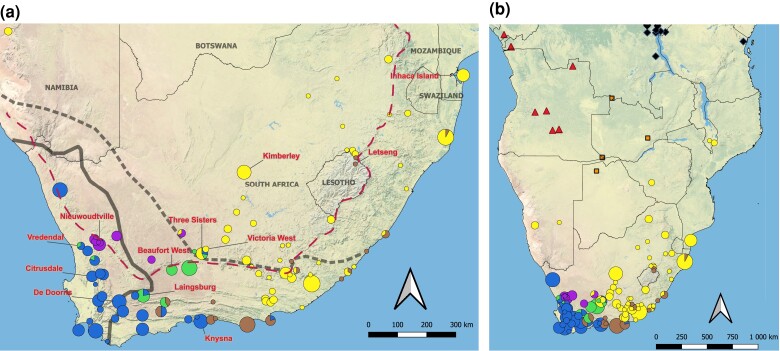
Geographical distribution of *X. laevis* sampling localities in (a) southern Africa used for RRGS (red labels) and Sanger sequencing of mitochondria (circular pies) and (b) sub-Saharan Africa with circles, triangles, squares, and diamonds for *X. laevis*, *X. petersii*, *X. poweri*, and *X. victorianus*, respectively). For *X. laevis*, colors correspond to mitochondrial clades in Fig. 5. In (a), solid and dotted lines indicate the margins of the winter rainfall zone to the southwest and the summer rainfall to the northeast, respectively (Chase and Meadows 2007) and a red dashed line indicates the Great Escarpment (see main text). The locality of the *X. gilli* sample, not shown, falls within the range of the winter rainfall (blue) clade of *X. laevis*, and is listed in Supplementary Table 2.

A survey of genetic variation in the nuclear genome was carried out using RRGS from 90 wild caught *X. laevis* individuals from 12 localities (Supplementary Table 1; Fig. 2a), one *Xenopus victorianus* from the Democratic Republic of Congo, three *Xenopus poweri* samples (one each from Nigeria, Cameron, and Botswana), and one *X*enopus *gilli* sample from South Africa. Genetic variation in the mitochondrial genome was characterized using Sanger sequencing of a ∼814 base pair portion of the mitochondrial 16S rRNA gene in a total of 455 samples from 183 localities (Fig. 2b), including 374 *X. laevis* samples from 183 localities (of which 105 were previously published; Furman *et al*. 2015), one *X. gilli* sequence that we defined as an outgroup based on previous analyses (Evans *et al*. 2004, 2019), and 41, 23, and 16 samples of *X. victorianus*, *X. poweri*, and *Xenopus petersii* samples (of which 41, 23, and 7, respectively, were previously published; Furman *et al*. 2015). We used primers 16Sc-L and 16Sd-H (Evans *et al*. 2003) for amplification and sequencing.

For the RRGS data, normalization and library preparation was performed at the Genomic Centre at the University of Laval using a double digest restriction enzyme associated DNA (Peterson *et al*. 2012) with *Sbf*I and *Msp*I restriction enzymes. The libraries were run on one third of lane of a NovaSeq S4 machine with paired end 150 base pair reads except for the *X. gilli* sample which had single-end reads. Reads were demultiplexed using Sabre (Joshi 2011), and trimmed using Cutadapt (Martin 2011) and Trimmomatic version 0.39 (Bolger *et al*. 2014).

RRGS reads were aligned to the *X. laevis* genome assembly version 9.2 (Session *et al*. 2016) using the mem function of BWA version 0.7.17 (Li and Durbin 2009) and SAMTOOLS version 1.10 (Li *et al*. 2009). Readgroups were added with PICARD version 2.23.3 (http://broadinstitute.github.io/picard/faq.html) and alignment and genotyping with the Genome Analysis Toolkit version 4.1.0.0 (McKenna *et al*. 2010) using the HaplotypeCaller, GenomicsDBImport, and GenotypeGVCFs functions. Data were then hard filtered using the VariantFiltration and SelectVariants functions of Genome Analysis Toolkit with filtering criteria recommended by best practices guide of the Broad Institute. Positions with the following attributes were removed: QD > 2.0, QUAL < 20, SOR > 3.0, FS > 60.0, MQ < 40.0, MQRankSum < −12.5, and ReadPosRankSum < −8.0, where these acronyms refer to variant confidence/quality by depth (QD), genotype quality (QUAL), Symmetric Odds Ratio of 2 × 2 contingency table to detect strand bias (SOR), Fisher exact test for strand bias (FS), map quality (MQ), a test for read map quality (MQRankSum), and a test for read position bias (ReadPosRankSum), respectively.

### Analyses of RRGS data

Geographical structure of genetic variation in each subgenome of *X. laevis* was visualized using principal components analysis (PCA) using the SNPRELATE (Zheng *et al*. 2012) package and ADMIXTURE (Alexander *et al*. 2009). These analyses were performed separately for variable positions in each subgenome using the RRGS data mapped to the whole genome after excluding unplaced scaffolds and excluding samples that were not *X. laevis*. For the principal components analysis, SNPs were pruned with a linkage disequilibrium threshold of 0.2 and requiring at least 50% of the samples to have called genotypes. The linkage disequilibrium threshold was selected based on comparisons to less stringent (0.4, 1) and more stringent (0) thresholds; the setting of 0.2 was selected as a compromise between maximizing the data included while also ensuring a high level of independence of each variable position. After pruning, a total of 59,998 and 47,203 positions were considered for the L and S subgenomes, respectively. Prior to ADMIXTURE analysis, the data were thinned by requiring at least 50% of the samples have called genotypes.

We quantified genetic exchange across an area where winter rainfall changes to summer rainfall, and biomes change from fynbos and succulent Karoo to Nama-Karoo (Chase and Meadows 2007) using Patterson's *D* statistic (Patterson *et al*. 2012). Patterson's *D* is calculated between three ingroup populations (P_1_, P_2_, P_3_) and an outgroup (O) whose evolutionary relationships follow this topology: (((P_1_, P_2_), P_3_), O). Positive values indicate an excess of shared sites between P_2_ and P_3_ (ABBA sites), whereas negative values indicate an excess of shared sites between P_1_ and P_3_ (BABA sites). We calculated Patterson's *D* using scripts in the genomics_general package (https://github.com/simonhmartin/genomics_general; Martin *et al*. 2015) with four non-independent parameterizations (comparisons 1–4) that were based on patterns of genome-wide similarity in the RRGS data discussed below. For these comparisons, populations were grouped based on inferred relationships among mitochondrial and autosomal DNA discussed below and listed in Supplementary Table 1. In addition, we performed a focused analysis on admixed individuals from Laingsburg relative to adjacent populations (Beaufort West, De Doorns, and Victoria West + Kimberley). Confidence intervals for Patterson's *D* were obtained using the block jackknife (Green *et al*. 2010). Patterson's *D* was calculated for each subgenome using the full data set, and also using a subset of each subgenome that included only genic regions (introns and exons). We performed these separate analyses with an aim of determining whether there might be distinctive, subgenome-specific patterns of gene flow in genic regions when compared with the whole genome. Because the amount of data differed substantially between the whole data set and the genic-only data set, we used 5 Mb genomic windows for the whole-genome data set and 10 Mb genomic windows for the genic-only data set. These sizes generally contained >100 ABBA and >100 BABA sites for each window for each analysis, which was deemed sufficient for calculations of confidence intervals using the block jackknife method described above.

A permutation test was used to evaluate the one-sided expectation that Patterson's *D* statistic should be more extreme for the S than the L subgenome. As discussed above, this expectation derives from the possibility of more locally advantageous variation in the L subgenome and the possibility of restorative gene flow in the S subgenome that could counteract the polyploid ratchet. The test statistic of the permutation tests was the absolute value of the weighted average *D* statistic of the S genome minus the absolute value of the weighted average *D* statistic of the L genome, with weighting based on the sum of the number of ABBA and BABA sites in each genomic window. This test statistic was compared with the distribution of 1,000 statistics that were obtained by permutating the L and S subgenome assignments of each window and then re-calculating the test statistic for each permutation.

Because the permutation tests generally found no significant difference between the L and S subgenome, we evaluated the power of our data to detect a difference. For each comparison, the standard error of Patterson's *D* of each subgenome was calculated following Busing *et al*. (1999). Quantile–quantile plots suggested that the distributions of Patterson's *D* within genomic blocks were approximately normally distributed within each subgenome (data not shown). The variance of Patterson's *D* was therefore estimated as the product of the squared standard error and the number of genomic blocks in each subgenome. The standard error of the test statistic is thus the square root of the sum of the within subgenome variances divided by the number of windows in each subgenome (Swinscow and Campbell 2002). We then calculated an estimated value of the test statistic where one would expect an 80% probability of detecting a significant difference, which is equal to 2.8 times the pooled standard error of the test statistic (Gelman and Hill 2006). This tells us how large the test statistic would have to be in order for the probability of type II error (failure to detect the null hypothesis of no difference when it is false) to be <20%. For each comparison, we also calculated the Cohen's *d* effect size (Cohen 2013), which is the difference between the mean divided by the pooled standard deviations, which we estimated as the square root of the average variance in the L and S subgenomes. The effect size measures the strength of the relationship between two variables (here Patterson's *D* and subgenome); values below 0.2 indicate a weak relationship (Cohen 2013).

### Phylogenetic analysis of mitochondrial and nuclear DNA

Because the population genetic relationships among the *X. laevis* autosomal data violate assumptions of a strictly bifurcating relationship among individuals, we summarized genetic distances between individuals using a neighbor-joining tree, which was calculated using Jukes–Cantor distances (Jukes and Cantor 1969) in PAUP* (Swofford 2002) and by constructing phylogenetic networks using the Neighbor-Net method (Bryant and Moulton 2004) using uncorrected *p*-distances. An input (nexus) file was generated from the genotypes using the vcf2phylip script (Ortiz 2019) and confidence of nodes was evaluated using 1,000 bootstrap replicates. We performed this analysis using variable positions only and requiring at least 80 out of the 95 samples to have called genotypes. This resulted in data sets with 1,018,084 and 801,859 variable positions for the L and S subgenomes, respectively.

We estimated evolutionary relationships among the partial mitochondrial sequences using the maximum likelihood criterion and the software IQ-TREE version 1.6.12 (Nguyen *et al*. 2015). The GTR + F + I + G4 model was used based on the Bayesian Information Criterion as implemented by IQ-TREE; confidence of nodes was evaluated using the ultrafast bootstrap approach (Minh *et al*. 2013). Additionally for the partial mitochondrial sequences, we constructed a parsimony network using TCS (Clement *et al*. 2000) and visualized with PopArt (Leigh and Bryant 2015).

## Results

### PCA and ADMIXTURE

**Fig. 3.**
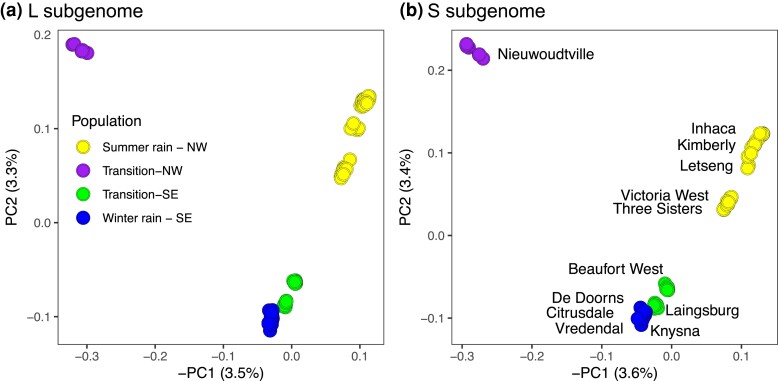
Principal components analysis of RRGS data from *X. laevis* for the (a) L and (b) S subgenomes. For each analysis, the percentages of genotypic variation represented by the first and second principal components (PC1 and PC2, respectively) are indicated. Patterns of population structure are similar in each subgenome and samples cluster by rainfall zone (indicated with color in the left panel) and by locality (labeled in right panel).

Analysis of the RRGS data using PCA and ADMIXTURE identified significant geographical structure to genetic variation, but patterns of population structure were almost identical in each subgenome. The PCA evidenced genetic differentiation within *X. laevis*, with the first and second principal component distinguishing from the summer, winter, northwest (NW) transitional rainfall zone, and the southeast (SE) transitional rainfall zone (Fig. 3), even though these components accounted for a small proportion of the total genotypic variation (∼7%). For both subgenomes in PC1, samples from the NW transition zone are more distinguished from populations in the summer or winter rainfall zones than are samples from the SE transition zone from populations in the summer or winter rainfall zones. In PC2, samples from the SE transition zone cluster closer to samples from the winter rainfall zone than the summer rainfall zone, whereas samples from the NW transition zone cluster closer to samples from the summer rainfall zone than the winter rainfall zone.

**Fig. 4.**
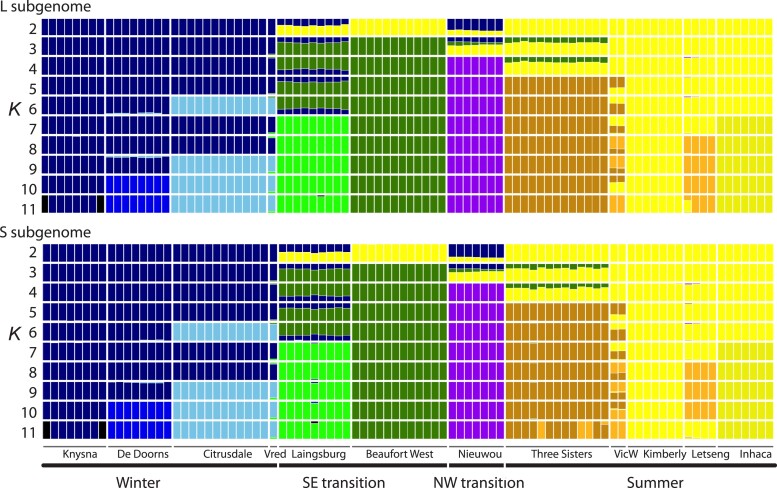
Admixture plot using only the L subgenome (top) or only the S subgenome (bottom) for 2–11 ancestry components (*K*) labeled on the left side. The order of the samples is from left to right matches the order of samples in Supplementary Table 1.

ADMIXTURE analysis recovered the most prominent structure between populations on either side of the southwest margin of the Great Escarpment (Figs. 2 and 4). This is apparent when *K* = 2 where samples to the southwest of this feature (Knysna, De Doorns, Citrusdale, and Vredendal) carry one ancestry component and those to the NW (Beaufort West, Three Sisters, Victoria West, Kimberly, Letseng, and Inhaca) carry another, and with admixed ancestry components in Laingsburg and Nieuwoudtville. At higher values of *K* additional population structure is evident, and patterns of population subdivision are similar to the PCA analysis. Based on the cross-validation procedure of ADMIXTURE (Alexander *et al*. 2009), the best value of *K* for these data is 5 or 6 for each subgenome (Supplementary Table 3). At *K* = 5, animals from Three Sisters are distinguished from their neighbors to the north (Victoria West, Kimberly, Letseng, and Inhaca) and those to the south (Knysna, De Doorns, Citrusdale, Laingsburg, and Beaufort West). At *K* = 6, Citrusdale and Vredendal are distinguished from the two other populations in the Winter rainfall region—Knysna and De Doorns.

These analyses also provide resolution into areas of admixture, such as *X. laevis* from Laingsburg, where most ancestry components are shared with individuals from Beaufort West which lies to the northeast of Laingsburg, but where individuals also carry an ancestry component that is most common in populations in the winter rainfall zone to the southwest. Similarly, *X. laevis* from Victoria West have an ancestry shared with *X. laevis* populations on the top of the Great Escarpment to Mozambique (Kimberly, Letseng, Inhaca) but also carry ancestry components from Three Sisters.

### No evidence for differential geneflow by subgenome

**Fig. 5.**
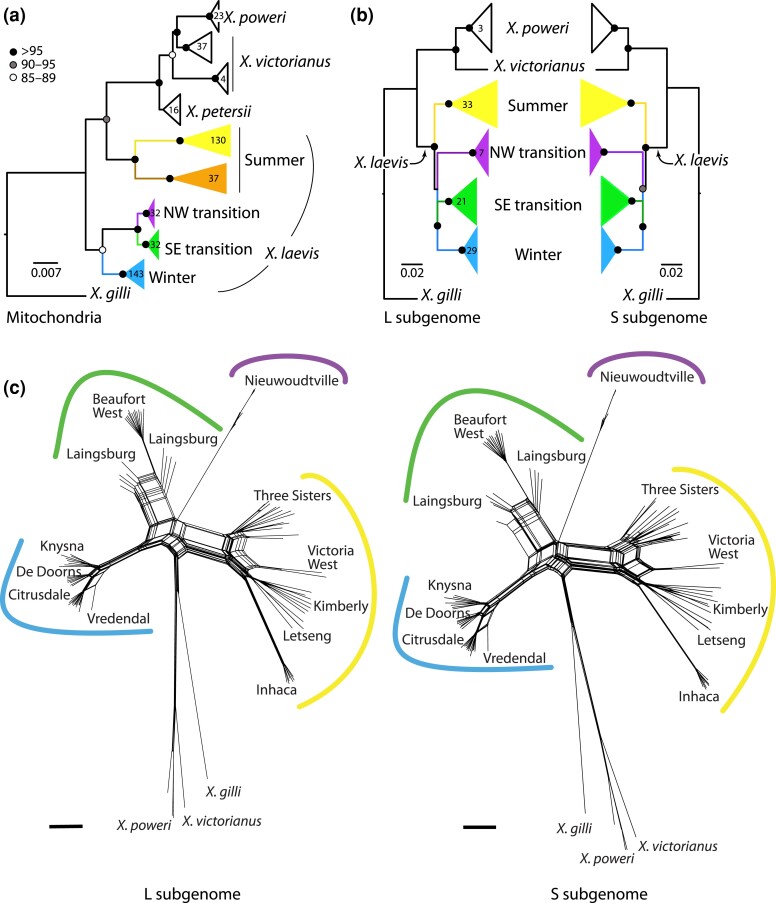
Evolutionary relationships among mitochondrial sequences (a) are different from (b and c) the L and S subgenomes. In (a) and (b), black, gray, and white nodes indicate bootstrap values as indicated on the scale; numbers inside clades indicate the sample sizes of individuals; samples sizes of the S subgenome (not shown) are identical to those for the L subgenome. The scale bar indicates substitutions per site for the mitochondrial phylogeny; branch lengths of the phenogram are scaled by only variable positions and are not comparable to mitochondrial branch lengths. Samples in each clade of the mitochondrial tree are listed in Supplementary Table 2. Samples with the brown mitochondrial lineage in (a) were not present in the RRGS data (b). In the Neighbor-Net networks in (c), black bars indicate 0.01 substitutions per site for each subgenome.

**Fig. 6.**
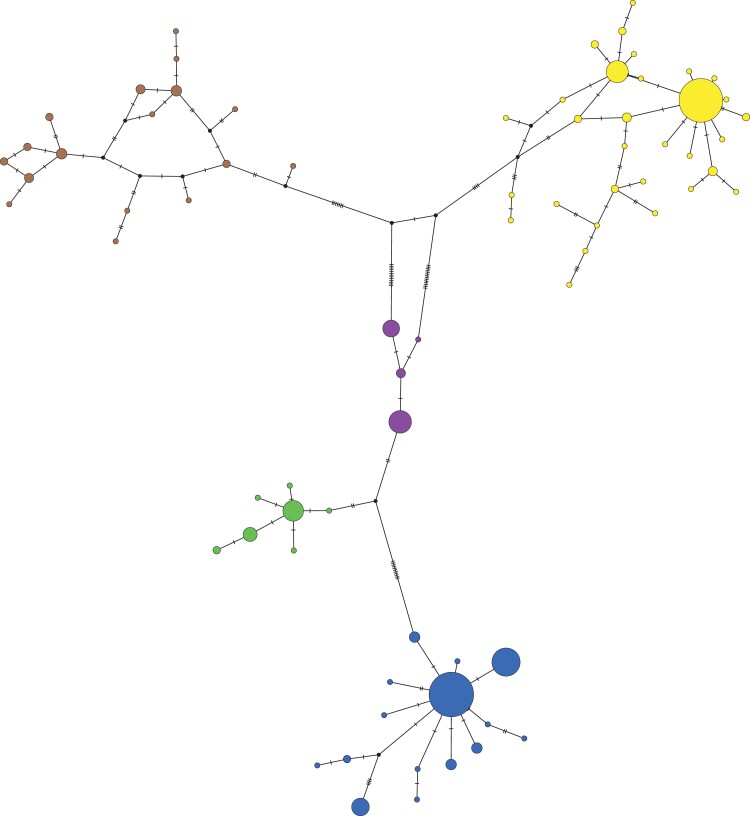
Parsimony network of partial *X. laevis* mitochondrial sequences. Colors correspond to Fig. 2; the size of the nodes is proportional to the number of samples with each haplotype. Inferred nodes are indicated with black nodes; hashes on branches indicate changes between nodes that are not represented by a sampled sequence.

We performed non-independent analyses of introgression based on Patterson's *D* statistic. For four of these five analyses (comparisons 1–4), we used the populations (winter, SE transition, NW transition, and summer) that were defined based on a combination of ecological and geological differences (Fig. 2) and genetic differentiation (Figs. 3–6). Some of these populations include data collected from individuals from multiple localities and, when more than one locality within a population was sampled, there is variation within each population in the geographic distances between localities. For these reasons, isolation by distance is expected to differently affect population structure within the summer, winter, and NW and SE transition zones due to differences in the size of each region. We therefore performed a fifth analysis that focused on the admixed population of Laingsburg as a way of exploring whether there might be subgenome effects at a smaller geographic scale (Laingsburg focus, Table 1). In this analysis, each of the three ingroup populations was sampled from only one location and we therefore do not expect isolation by distance within each population to be substantial. Admixture in Laingsburg is evidenced by ADMIXTURE analysis with *K* = 5 (Fig. 4) and by Sanger sequences from autosomal and mitochondrial DNA (Du Preez *et al*. 2009; Furman *et al*. 2015).

**Table 1.** Patterson’s *D* statistic and 95% confidence intervals (95% CIs) for each subgenome for four comparisons within *X. laevis* involving populations grouped by rainfall pattern (Winter, NW transition, SE transition, Summer, and the *X. victorianus* + *X. poweri* outgroup (Out) and a focused analysis of the zone of admixture in Laingsburg. *P*-values of permutation tests (*P*) indicate that the *D* statistic is not significantly different between the L and the S subgenome for any comparison. For each comparison and for each subgenome the number of ABBA and BABA sites (nABBA, nBABA, defined in main text) is indicated. For each comparison the test statistic, pooled standard deviation, value of the test statistic that would deliver 80% power, *P*-value, and effect size is also provided. The effect size of comparisons with a test statistic below zero is not shown. As detailed in Fig. 4, the winter population includes samples from Knysna, De Doorns, Citrusdale, and Vredenburg; the SE transition includes samples from Laingsburg and Beaufort West; the NW transition zone includes samples from Nieuwoudtville; the summer population includes samples from Three Sisters, Victoria West, Letseng, and Inhaca.

P1, P2, P3, O	nABBA	nBABA	*D* (95% CI)	Test statistic	Pooled standard deviation	Value of test statistic to have 80% power	*P*-value	Effect size
Comparison 1: winter, SE transition, summer, out								
ȃL subgenome	38,408	20,603	0.302 (0.291–0.312)	<0	0.074	0.206	1.000	-
ȃS subgenome	25,342	19,882	0.121 (0.113–0.129)					
Comparison 2: winter, NW transition, summer, out								
ȃL subgenome	33,283	25,295	0.136 (0.127–0.145)	0.006	0.081	0.227	0.214	0.072
ȃS subgenome	28,799	21,628	0.142 (0.131–0.153)					
Comparison 3: SE transition, NW transition, summer, out								
ȃL subgenome	27,614	38,789	−0.168 (−0.177 to −0.160)	<0	0.077	0.216	1.000	-
ȃS subgenome	23,752	32,632	−0.158 (−0.168 to −0.147)					
Comparison 4: winter, SE transition, NW transition, summer								
ȃL subgenome	2,994	5,762	−0.316 (−0.335 to −0.297)	<0	0.160	0.447	1.000	-
ȃS subgenome	2,793	5,366	−0.315 (−0.339 to −0.291)					
Laingsburg focus: Laingsburg, Beaufort West, De Doorns, Victoria West + Kimberly								
ȃL subgenome	20,320	38,782	−0.312 (−0.326–−0.299)	<0	0.116	0.324	1.000	-
ȃS subgenome	17,910	32,609	−0.291 (−0.306–−0.276)					

For comparisons 1–4, Patterson's *D* was significantly different than zero for both subgenomes but this statistic was not significantly different between subgenomes within each comparison (Table 1). The weighted average of Patterson's *D* was more extreme (but not significantly so) in the S than the L subgenome only for comparison 2. Overall, this is consistent with extensive, geographically structured gene flow, but does not provide evidence for differential gene flow by subgenome in most comparisons on a genome-wide scale. For all four comparisons, the effect size is below 0.2, suggesting that the effect of subgenome on Patterson's *D* is weak (Table 1). The test statistics are generally far smaller than the value estimated to have 80% power to detect a significant difference. This indicates that the effect of subgenome on Patterson's *D* would have to be far larger in order for this effect to be detected with these data. When only genic areas were considered, results were similar to the analysis of all RRGS data within each subgenome, with three of four comparisons having more extreme values of Patterson's *D* statistic in the L rather than the S subgenome, and with the values in comparison 2 being not significantly different (Supplementary Table 4).

For comparison 1, Patterson's *D* is positive for both subgenomes, supporting the geographically based intuition that there is more gene flow between the summer rainfall population and the SE transition population than between the summer rainfall population and the winter rainfall population. Similarly, for comparison 2 Patterson's *D* is also positive, indicating more gene flow between the summer rainfall population and the NW transition population than between the summer rainfall population and the winter rainfall population, which is also unsurprising since the NW transition population is closer than the winter rainfall population to the summer rainfall population. For comparison 3, Patterson's *D* is negative, indicating more gene flow between the summer rainfall population and the SE transition population than between the summer rainfall population and the NW transition population. This result is consistent with the PCA and admixture analyses discussed above, which highlight the distinctiveness of the NW transition population. For comparison 4, Patterson's *D* is negative, indicating more geneflow between the NW transition population and the winter rainfall population than between the NW transition population and the SE transition population. This suggests that gene flow within the transition zone is more limited than between the transition zone and the neighboring regions, a finding that is further highlighted by generally non-overlapping mitochondrial clades as detailed below.

For both subgenomes, Patterson's *D* was negative for the Laingsburg focus comparison, which indicates more genetic exchange between the geographically proximal populations in De Doorns and Laingsburg when compared with the more distant comparison between De Doorns and Beaufort West. When only genic regions were considered, there also was a more extreme (negative) Patterson's *D* statistic in the L rather than the S subgenome (Supplementary Table 4).

Subgenome-wide genetic similarity as summarized by phenograms also suggested a high degree of congruence between the L and S subgenomes (Fig. 5, b and c). Phenetic relationships among nuclear variation are similar in each subgenome and indicate higher similarity within *X. laevis* individuals compared with between *X. laevis* and either *X. victorianus* or *X. poweri* (Fig. 5, b and c). Within *X. laevis*, similarity within each population (winter rainfall, SE transition, NW transition, summer rainfall; Table 1) is higher than between these populations (Fig. 5, b and c).

### Phylogeography of *X. laevis* in South Africa

Major topographic relationships recovered from analysis of mitochondrial and nuclear DNA (Fig. 5) are consistent with previous analyses of fewer partial mitochondrial sequences or concatenated nuclear loci (Furman *et al*. 2015). These analyses suggest that mitochondrial DNA within *X. laevis* is paraphyletic relative to *X. victorianus*, *X. poweri*, and *X. petersii*, a result that is discordant with phenetic analysis of nuclear DNA which finds lower intraspecific than interspecific divergence in both subgenomes (discussed above). Mitochondrial sequences support a sister relationship between the NW and SE transition zones; this relationship was unresolved in the phenetic analysis of each subgenome (Fig. 5).

The summer rainfall clade covers most of the range of *X. laevis* in southern Africa, including populations sampled in South Africa, Namibia, Zimbabwe, Malawi, and Mozambique. Increased sampling identified a previously unreported mitochondrial clade (indicated in brown in Fig. 5a) in the summer rainfall region. This clade is prevalent in the southeastern portion of the distribution of *X. laevis* east of the Keurbooms River and is also found at lower frequencies in animals that were sampled north of this area in Letseng, where the previously reported clade in the summer rainfall region is common.

New sampling of mitochondrial sequences also permitted us to identify the geographic margins of previously identified mitochondrial clades. For example, the NW transition zone clade, previously known only from Nieuwoudtville at the northern extent of the Cape Fold Mountains (Furman *et al*. 2015), extends east into the Karoo (Fig. 2). The SE transition zone clade, which was previously known from Laingsburg and Beaufort West (Du Preez *et al*. 2009; Furman *et al*. 2015), also occurs on the inner margins of the Cape Fold Mountains, mainly within the Karoo basin. The summer rainfall clade encompasses all populations of *X. laevis* away from a southwestern portion of southern Africa, including Namibia, Zimbabwe, Mozambique, Lesotho, Eswatini and Malawi. The winter rainfall clade is carried by animals from the southwestern extreme of the distribution of *X. laevis* and at least as far north as Springbok, and also extends into low lying regions in the southern portion of the transition zone, with the eastern margin at the Keurbooms River.

Multiple mitochondrial clades were observed in contact zones between most pairs of clades (Supplementary Table 1). This geographic comingling of mitochondrial clades at the margins of their distributions corresponds with evidence of multiple ancestry components in autosomal DNA from the same individuals from at least two localities (Vredendal, Three Sisters).

Because population dynamics of mitochondrial DNA may not be fully captured by phylogenetic analyses that assume strictly bifurcating relationships, we also constructed a network of relationships among the partial mitochondrial sequences (Fig. 6). This network identified one star-shaped relationship in the winter (blue) clade and two in the summer (yellow) clade that are suggestive of population expansion (Avise 2009; Ferreri *et al*. 2011), whereas the comparatively more missing/inferred haplotypes in the brown clade suggests a more stable or contracting population. Relatively few changes separate the green and purple clades, which is consistent with the close inferred phylogenetic relationship between these clades (Fig. 5).

## Discussion

*Xenopus laevis* has an unusual relationship with humans, first as a pregnancy test (Weisman and Coates 1941), and later as a model organism for developmental biology (Cannatella and de Sá 1993; Gurdon 1996; Harland and Grainger 2011). Historical records demonstrate that exported individuals were sourced from multiple localities in southern Africa (Van Sittert and Measey 2016), and some invasive populations are admixed (De Busschere *et al*. 2016; Measey *et al*. 2017). Within its native range, *X. laevis* is the most widely distributed of all amphibian species in South Africa, with records from nearly every quarter degree square (Measey 2004). This species has been the focus of several previous genetic and phylogeographic studies (Grohovaz *et al.* 1996; Evans *et al.* 1997, Measey and Channing 2003; Evans *et al.* 2004; Du Preez *et al.* 2009; Furman *et al.* 2015, 2017), but here we show that the diversity is greater than previously appreciated, including the discovery of a novel mitochondrial clade. Despite previous studies suggesting the potential for populations to be structured at the subgenome level (Wei *et al*. 2016; Kryvokhyzha *et al*. 2019), we find no evidence of the polyploid ratchet or for differential geneflow in each subgenome. This study, therefore, provides a hitherto unappreciated example of a tetraploid species with substantial phylogeographic structure but without substantial independent genomic structure in each subgenome.

The substantial and complex population structure of *X. laevis* coupled with its wide use as an experimental organism and new role as an invasive species, underscore the importance of understanding genetic variation in natural populations and how this relates to diversity in captive and invasive populations.

### Absence of a substantial subgenome effect for most comparisons

Subgenome evolution is not universally asymmetric (e.g. Liu *et al*. 2017; Wu *et al*. 2021) and the effect of subgenome on various genomic characteristics (gene flow, gene expression, etc.) may be modest, species-specific, temporally dynamic, or centered only on small genomic regions.

Our analyses of genetic similarity, population structure, and introgression failed to detect significant or substantial differences between the L and S subgenomes in natural populations of *X. laevis* in multiple comparisons with all the RRGS data, and in multiple comparisons with RRGS data from genic regions. In fact, for four of five comparisons in each category (all RRGS data or genic regions only), Patterson's *D* statistic was more extreme (positive or negative) for the L subgenome than the S subgenome; for the fifth comparison (comparison 2 in both cases) there was no significant difference between the L and S subgenomes. Overall, these findings are inconsistent with the possibility that gene flow in the S subgenome is atypically high in order to mitigate deleterious effects of the polyploid ratchet.

As discussed above, subgenomes do have significant and substantial effects on several other aspects of *X. laevis* genome evolution, including transposable element mobility and composition, genomic rearrangements, gene silencing, expression, and length, and the strength of purifying selection on genic regions (Session *et al*. 2016; Elurbe *et al*. 2017; Furman *et al*. 2018). One possibility is that differential introgression within subgenomes could occur in small genomic regions that we lacked statistical power to discern. An interesting question that could be explored with additional high-quality genome assemblies from other allotetraploid *Xenopus* species (in subgenus *Xenopus*) asks whether and how the rate of genomic rearrangements has varied over time since allotetraploidization. One possibility is that purifying selection counteracted the polyploid ratchet by eliminating rearrangements that occurred recently. If this were the case, many rearrangements in the S subgenome of *X. laevis* (Session *et al*. 2016) would be shared with other allotetraploid species that diversified soon after allopolyploidization.

### Genetic differentiation of the nuclear genome of *X. laevis*

Previously, patterns of population structure within the natural range of *X. laevis* were inferred from genetic variation in portions of the mitochondrial genome and 2–15 nuclear genes (Grohovaz *et al*. 1996; Evans *et al*. 1997; Measey and Channing 2003; Du Preez *et al*. 2009; Furman *et al*. 2015). These efforts identified significant intraspecific differentiation, and admixture in a population from Laingsburg from source populations to the southwest and northeast. The far larger sample of genetic variation afforded by RRGS data examined here further contextualizes the geographic distribution of genetic variation in *X. laevis*, including the distribution of ancestry components at two localities (Citrusdale and Three Sisters) that have not previously been studied.

Our results are consistent with Furman *et al*. (2015) in identifying substantial population structure in *X. laevis* from South Africa (including Lesotho), particularly between individuals sampled near Nieuwoudtville, which is within the NW transition zone. The distribution of differentiated populations roughly matches the distribution of mitochondrial clades (purple, yellow, blue, green respectively). In the RRGS data, there were no samples that carried the brown clade, so we were unable to assess genetic variation in their nuclear genomes. Patterson's *D* provides geographically plausible inferences of gene flow between regions, including higher levels of gene flow between geographically adjacent compared with non-adjacent areas.

Analysis of genetic variation in the nuclear genome further characterized several zones of admixture, including one that was previously identified (Laingsburg; Du Preez *et al*. 2009), and one that was previously studied with less information and whose ancestry components were incompletely characterized (Victoria West; Furman *et al*. 2015). In Laingsburg, ancestry of individuals was primarily shared with Beaufort West (which lies to the northeast of Laingsburg) but also with a component from the winter rainfall zone to the southwest of Laingsburg. Previously uncharacterized genetic variation from Three Sisters, which lies between Beaufort West and Victoria West, highlights a gradient of ancestry components as one moves through the transitional zone (Fig. 2) from southwest to northeast toward the Karoo. New information from Citrusdale and Vredendal confirms that the winter rainfall population occurs throughout this region.

New samples analyzed here from near Sentinel Peak (Letseng), a high altitude area in the Drakensberg, which is >3,000 m above sea level (asl), were not substantially differentiated from a population near Kimberly, which is ∼1,100 m asl. This suggests that genome-wide genetic changes associated with adaptation to high altitude (see Wagener *et al*. 2021) are small in comparison to population structure within *X. laevis*.

### Mitochondrial variation: margins of clades

The first phylogeographic exploration of mitochondrial variation in *X. laevis* showed the existence of the winter rainfall clade (blue), the summer rainfall clade (yellow), and an isolated sample from the NW transition zone—Nieuwoudtville (purple) as a divergent clade (Grohovaz *et al*. 1996). Subsequent phylogeographic studies also found the NW transition clade in and around Nieuwoudtville (Measey and Channing 2003; Furman *et al*. 2015). Here we show that this clade is also carried in animals east of Nieuwoudtville and into the Karoo. Nieuwoudtville is thus at the western edge of the distribution of this clade, with animals from the winter rainfall clade being found at similar altitude immediately south, and to the west. Our sampling was unable to characterize the northern extent of the NW transition clade, as this area is especially arid and contains the least records in the region for this species (Measey 2004).

Inferred relationships between the mitochondrial sequences differ in several ways from patterns of genome-wide similarity in the nuclear genome, including within *X. laevis* and between *X. laevis* and other closely related species. Ancestral polymorphism is a plausible explanation for these discrepancies. Male-biased migration contributes to mitonuclear phylogenomic discordance in some species, but there is no evidence for this in *X. laevis* (Measey 2016; Courant *et al*. 2017; De Villiers and Measey 2017; Louppe *et al*. 2020).

Areas for further investigation include (1) the limit of the winter rainfall (blue) clade in the far Northern Cape, which may be the Fish River at the border with Namibia based on findings in other amphibians (see Tolley *et al.* 2014), (2) the northern extent of the purple clade which could be bounded by the arid regions that straddles the border between South Africa and Botswana near Namibia, and (3) the eastern limit of the green clade, which may extend into the eastern Karoo region.

### Conclusion

Genetic variation from the nuclear genome of an allopolyploid frog—*X. laevis—*in their natural habitat failed to identify substantial differences in population structure or gene flow in each subgenome of this allotetraploid species. These results are inconsistent with the proposal that a polyploid ratchet favors restorative gene flow in the S subgenome—at least on a scale that is substantial enough to detect using the RRGS data we collected. This suggests that the effect of subgenomes on gene flow and population structure is either small or absent across the genome of *X. laevis*. Population structure in the nuclear genome includes several zones of admixture, and corresponds roughly with distributions of mitochondrial clades, ecological and rainfall transition zones, and topographic relief associated with the Cape Fold Mountains, although surprisingly less so for the Drakensberg Mountains.

## Supplementary Material

jkac325_Supplementary_Data

## Data Availability

The RRGS data from this study are listed in Supplementary Table 1 and have been deposited in the Short Read Archive of NCBI (BioProject PRJNA906487). All new Sanger sequences are listed in Supplementary Table 2 and have been submitted to GenBank (accessions: OP902610–OP902889). Supplemental material available at G3 online.

## Notes

*Communicating editor: J. Wendel*
